# Research advances in the study of traditional Chinese medicine formula granules on signaling pathway-mediated disease mechanisms

**DOI:** 10.3389/fphar.2025.1609211

**Published:** 2025-06-03

**Authors:** Zhuo-Xu Gu, Zhong-Hao Wang, Xian-Quan Zhang, Guang-Hui Zhou, Gui-Hong Liang, Ling-Feng Zeng, Jun Liu

**Affiliations:** ^1^ State Key Laboratory of Traditional Chinese Medicine Syndrome/The Second Clinical College of Guangzhou University of Chinese Medicine, Guangzhou, China; ^2^ Guangdong Provincial Key Laboratory of Chinese Medicine for Prevention and Treatment of Refractory Chronic Diseases/The Second Affiliated Hospital of Guangzhou University of Chinese Medicine (Guangdong Provincial Hospital of Chinese Medicine), Guangzhou, China; ^3^ Bone and Joint Research Team of Degeneration and Injury, Guangdong Provincial Academy of Chinese Medical Sciences, Guangzhou, China; ^4^ Guangdong Second Traditional Chinese Medicine Hospital (Guangdong Province Engineering Technology Research Institute of Traditional Chinese Medicine), Guangzhou, China

**Keywords:** traditional Chinese medicine formula granules, signaling pathways, mechanisms of disease, secondary metabolites, orthopedic diseases, plant-based natural products

## Abstract

As a modern dosage form of traditional Chinese medicine, Traditional Chinese Medicine Formula Granules (TCMFG) maximally retains active metabolites through standardized production processes, including dynamic countercurrent extraction and low-temperature concentration. This serves as a critical material basis for elucidating its multi-target regulatory mechanisms. Recent studies have highlighted the significant potential of TCMFG in treating complex diseases, such as inflammation, tumors, metabolic disorders, fibrosis, and orthopedic conditions like osteoarthritis and osteoporosis, by modulating signaling pathways such as TLR4/NF-κB, MAPK, NLRP3 inflammasome, PI3K/Akt/mTOR, Wnt/β-catenin, and TGF-β/Smads. For example, berberine alleviates acute lung injury by inhibiting the TLR4/NF-κB pathway, while tetramethylpyrazine derived from *Ligusticum chuanxiong* Hort granules mitigate joint destruction in rheumatoid arthritis by suppressing MAPK signaling. Curcumin analogs inhibit tumor metabolic reprogramming via the PI3K/Akt pathway. Despite these advances, current research encounters challenges, such as single-component analysis, unclear synergistic mechanisms of compound mixtures, and insufficient clinical evidence for translational applications. In the future, integrating multi-omics technologies (e.g., metabolomics, spatial transcriptomics) will be crucial for exploring supramolecular synergistic effects of compound mixtures. Furthermore, large-scale clinical trials are necessary to validate its potential in precision medicine. This article systematically reviews the progress of TCMFG research in signaling pathway-mediated disease mechanisms and outlines future directions to enhance its application in modern precision medicine.

## 1 Introduction

Traditional Chinese Medicine Formula Granules (TCMFG) also categorized as Chinese herbal concentrated granules, are produced through a series of standardized processes including water extraction, separation, concentration, and drying of individual Chinese herbal decoction pieces. The raw materials used are herbal slices rather than crude herbal materials, which aligns with the theoretical framework of traditional Chinese medicine’s syndrome differentiation and treatment (Lu et al., 2022; Zhu et al., 2024). In comparison to traditional decoctions, their production involves a highly controlled process (e.g., precise regulation of extraction temperature, solvent concentration, drying time, and other parameters), ensuring the stability and batch-to-batch consistency of active metabolites. This provides a solid material foundation for modern pharmacological research. In recent years, studies on the signaling pathways modulated by single Chinese herbal concentrated granules or individual metabolites have elucidated various disease mechanisms, including inflammation, tumors, fibrosis, and neurodegenerative disorders.

## 2 Production technology and advantages of TCMFG

TCMFG strictly adheres to the “Guidelines for the Development of Single Chinese Medicine Concentrated Granules,” utilizing Chinese medicine decoction pieces that comply with processing specifications as raw materials. Through advanced modern processes, including whole-component water extraction, low-temperature concentration, spray drying, and volatile oil preservation technology, TCMFG ensures the maximal retention of active metabolites (Lu et al., 2022). Studies have demonstrated that the application of dynamic countercurrent extraction coupled with low-temperature concentration effectively maximizes the retention of active metabolites in Chinese herbal decoction pieces. Additionally, this approach ensures a utilization rate of medicinal materials ranging from 60% to 70% (Yin et al., 2022). For certain drug toxicities, several studies have indicated that the incidence of adverse reactions associated with the use of Chinese herbal decoction pieces of aconite is higher compared to TCMFG. This may be attributed to the extraction method employed in TCMFG production, which could potentially reduce the concentration of aconitine, a toxic component found in aconite (Yang et al., 2018). In addition, the processes of metabolite extraction and labeling, as well as the standardized evaluation of TCM compound granules, were presented in tabular form (Table 1).

**TABLE 1:** Standardization of the labeling process for the extraction of TCM metabolites

TCM metabolites	Extraction process	Standardized marker	References
*Berberine*	Dynamic countercurrent extraction combined with low temperature concentration technology	Maximum retention of active metabolites, reduced concentration of toxic metabolites such as aconitine, batch to batch consistency	(Su et al., 2019), (Wang et al., 2024b), (Li et al., 2023)
*Tetramethylpyrazine*	Whole-component water extraction combined with low-temperature concentration technology	The stability of the active metabolites is ensured by standardized processes, including essential oil preservation techniques	Li et al. (2019a)
*Glycyrrhizic acid*	Acid-base neutralization reaction	Synergistic attenuation and synergism were used to optimize toxicity control in compound decoction	Ding et al. (2023)
*Astragalus polysaccharides*	Low-temperature dynamic countercurrent extraction with spray drying	Batch to batch consistency, synergistic supramolecular structure of polysaccharide and alkaloid (e.g., MGF-SA)	(Yang et al., 2024a; Huang et al., 2024)
*Curcumin*	Water extraction was combined with low-temperature spray drying	Standardized production ensures stability of active metabolites, such as curcumin analogues, and clinical dosage control	Jin et al. (2022)
*Artemisinin*	Whole component extraction with low-temperature concentration technology	Heat-sensitive metabolites are retained and oxidative degradation is reduced by optimizing the process	Wang et al. (2024a)
*Panax notoginseng saponins*	Dynamic countercurrent extraction combined with volatile oil preservation technology	Multi-component co-regulation (e.g., inhibition of TGF-β/Smads and PDGF/ERK pathways)	Wu et al. (2021)
*Paeoniflorin*	Low-temperature water extraction with spray drying	Stability of the active ingredient reduces hepatotoxicity (e.g., lowers ALT/AST levels)	Deng et al. (2024)
*Baicalin*	Dynamic countercurrent extraction at low temperature and hypoxic treatment	Supramolecular complex formation, such as synergistic inhibition of the NLRP3 inflammasome with scutellaria	(Jin et al., 2019), (Liu et al., 2021)
*Ginsenoside Rg1*	Water extraction and low-temperature concentration of whole metabolites	Standardized production ensures bioavailability and reduces oxidative effects	Qin (2006)
*Tanshinone IIA*	Low-temperature extraction with dynamic countercurrent concentration	The activity of mitophagy-related proteins (PINK1 and Parkin) was optimized, and the clinical dosage was standardized	(Si et al., 2023), (Jin et al., 2022)
*Liquidambaric acid*	Water extraction combined with low-temperature concentration	Direct TRAF2 targeting inhibits the Wnt/β-catenin pathway and ensures batch stability	Yan et al. (2022)
*Gallic acid*	Water extraction and low-temperature spray drying	Wnt/β-catenin pathway inhibition for standardized treatment of gastric precancerous lesions	Liao et al. (2023)

## 3 Regulatory mechanisms of TCMFG active metabolites on signaling pathways

### 3.1 Pathways related to anti-inflammatory and immune regulation

#### 3.1.1 TLR4/NF-κB signaling pathway

This pathway serves as the “core switch” in the inflammatory cascade and is implicated in a wide range of inflammatory diseases, including acute pancreatitis (AP) and sepsis (Kuzmich et al., 2017; Abdelmageed et al., 2021).

The TLR4/NF-κB signaling pathway functions as a pivotal “core switch” in the inflammatory cascade and is associated with various inflammatory diseases. *Berberine* (BBR), the primary active ingredient in *Coptis rhizoma* concentrate granules, inhibits LPS-induced NF-κB activation by competitively binding to the MD2 domain of TLR4, thereby downregulating proinflammatory cytokines such as TNF-α and IL-6 (Su et al., 2019). Notably, BBR demonstrates efficacy comparable to dexamethasone in treating acute lung injury; for instance, a dose of 50 mg/kg BBR reduced alveolar macrophage infiltration by 60%. Recent studies emphasize the distinct advantages of TCMFG over single-component formulations. For example, when combined with *Scutellaria baicalensis polysaccharides* in a compound formulation, BBR exhibits enhanced bioavailability (AUC increased by 1.8-fold) and significantly reduced hepatotoxicity (ALT levels decreased by 35% compared to BBR alone) (Wang K. et al., 2024). This synergistic effect is attributed to the stabilization of BBR in the gastrointestinal tract mediated by polysaccharides and dual modulation of both the TLR4/NF-κB and Nrf2 pathways. Moreover, clinical trials reveal that compound granules containing BBR and *Astragalus membranaceus* saponins achieve a 42% reduction in TNF-α levels in rheumatoid arthritis patients, surpassing the efficacy of BBR monotherapy (28% reduction) (Li et al., 2023). These findings highlight the essential role of multi-component interactions in enhancing therapeutic outcomes while minimizing adverse effects.

#### 3.1.2 Mitogen-Activated Protein Kinase (MAPK) signaling pathway

The MAPK pathway is a critical signal transduction pathway ubiquitously present in eukaryotic cells. It plays a pivotal role in regulating various biological processes, including cell growth, differentiation, stress response, and apoptosis. This pathway primarily comprises three subfamilies: Extracellular Signal-Regulated Kinase (ERK), c-Jun N-terminal Kinase (JNK), and p38 MAPK, which are involved in modulating inflammatory responses and apoptosis (Liu et al., 2016; Delavari et al., 2014; Fang and Richardson, 2005). *Ligusticum chuanxiong* Hort concentrated granules contain *tetramethylpyrazine*, which exhibits partial inhibition of JNK and p38 phosphorylation while exerting minimal influence on ERK activity. In the rheumatoid arthritis (RA) model, *tetramethylpyrazine* (40 mg/kg) significantly decreased MMP-9 expression in synovial cells, thereby delaying joint destruction (Li Y. et al., 2019). In addition, *Forsythia suspensa Granules* suppressed Th17 cell differentiation by inhibiting the p38 MAPK/STAT3 signaling pathway and significantly decreased IL-17A secretion (with an inhibition rate of 68.3%) in the psoriasis model (Lin et al., 2022). *Paeoniflorin* significantly decreased the serum levels of ALP, γ-GT, AST, TBIL, and ALT in APAP-induced mice, while mitigating liver tissue inflammation and edema. These findings suggest that *paeoniflorin* exerts its protective effects by activating autophagy and suppressing oxidative stress and apoptosis via the MAPK/mTOR signaling pathway, thereby alleviating hepatic injury (Deng et al., 2024).

#### 3.1.3 NLRP3 inflammasome pathway

The NLRP3 inflammasome can be triggered by a diverse array of pathogen-associated molecular patterns (PAMPs) and damage-associated molecular patterns (DAMPs). Upon activation, NLRP3 undergoes oligomerization and subsequently activates caspase-1. The activated caspase-1 further facilitates the maturation and secretion of pro-IL-1β and pro-IL-18, two critical cytokines involved in orchestrating inflammatory responses. The activation and regulation of the NLRP3 inflammasome encompass a multitude of signaling pathways, post-translational modifications, and interactions with partner molecules that modulate its function (Swanson et al., 2019; Blevins et al., 2022). NLRP3 inflammasome dysfunction is involved in a variety of diseases, such as liver disease, nephropathy, neurological disease, and diabetes. *Baicalin* markedly suppressed the upregulation of TLR4 expression, the phosphorylation and degradation of IκBα, as well as the subsequent nuclear translocation of NF-κB p65. Consequently, the inhibitory effect of *baicalin* on NLRP3 inflammasome activation is mediated via the downregulation of TLR4/NF-κB signaling pathway. These findings suggest that *baicalin* holds great promise as a neuroprotective agent for the prevention and management of microglia-mediated neuroinflammation in the progression of Alzheimer’s disease (Jin et al., 2019). *Isochlorogenic acid A*, a natural compound formed via the esterification and condensation of quinic acid and caffeic acid, is predominantly found in Lonicerae japonica. *Isochlorogenic acid A* significantly suppresses the expression of NLRP3, caspase-1, NF-κB p65, phosphorylated NF-κB p65 (p-NF-κB p65), phosphorylated IκB (p-IκB), and RANKL in synoviocytes derived from collagen-induced arthritis rats. Furthermore, it downregulates plasma levels of IL-1β, IL-6, TNF-α, CRP, IFN-γ, and IL-18, which contributes to the reduction of toe swelling in rats. These findings indicate that Isochlorogenic acid A exhibits potent anti-inflammatory effects on collagen-induced arthritis, with its activity potentially attributed to the inhibition of NLRP3 inflammasome activation and the modulation of NF-κB phosphorylation (Liu et al., 2021). *Artemisinin* decreased the production of reactive oxygen species (ROS), attenuated the mitochondrial membrane potential (Δψm), inhibited pyroptosis, preserved neuronal cell morphology, and downregulated the levels of IL-1β and IL-18 as well as the expression of NLRP3, ASC, Caspase-1, and TXNIP, thereby mitigating neuronal pyroptosis induced by ischemic stroke (Wang Y. et al., 2024).

### 3.3 Metabolic and fibrosis-related pathways

#### 3.3.1 AMPK pathway

The classical role of AMP-activated protein kinase (AMPK) is as a cellular energy sensor that is activated in response to decreases in cellular energy status, signaled by an increase in the AMP-to-ATP and ADP-to-ATP ratios. Upon activation, AMPK restores energy homeostasis by promoting catabolic pathways that generate ATP while simultaneously inhibiting processes that consume ATP (Steinberg and Hardie, 2023).

Nonalcoholic fatty liver disease (NAFLD), particularly nonalcoholic steatohepatitis (NASH), has emerged as a predominant cause of liver transplantation and liver-related mortality. NASH represents the hepatic manifestation of metabolic syndrome and is defined by the presence of hepatic steatosis, inflammation, hepatocellular injury, and fibrosis of varying severity. *Cordycepin* exhibits a substantial protective effect against hepatic steatosis, inflammation, liver injury, and fibrosis in mice under metabolic stress through the activation of the AMPK signaling pathway. Additionally, cordycepin may serve as a potential AMPK activator for the treatment of NASH (Lan et al., 2021). *Ginsenoside RK1* modulates the phosphorylation of AMP-activated protein kinase (AMPK) and its downstream target, NF-E2-related factor 2 (Nrf2), thereby optimizing mitochondrial membrane potential, reducing reactive oxygen species (ROS) levels, and alleviating Alzheimer’s disease (AD)-like pathology (She et al., 2024). It was demonstrated (Si et al., 2023) that *Tanshinone IIA* (Tan IIA) significantly enhanced the viability of HT22 cells under intermittent hypoxia (IH). Treatment with Tan IIA improved mitochondrial membrane potential, decreased apoptosis rates, attenuated oxidative stress, and promoted autophagy in HT22 cells exposed to IH conditions. Furthermore, Tan IIA increased the phosphorylation of AMPK as well as the expression levels of LC3II/I, Beclin-1, Nrf2, HO-1, SOD2, and Bcl-2/Bax. Additionally, Tan IIA reduced the phosphorylation of mTOR and downregulated the expression of NOX2 and cleaved caspase-3/caspase-3. The neuroprotective mechanism of Tan IIA may primarily involve the inhibition of oxidative stress and neuronal apoptosis through the activation of the AMPK/mTOR autophagy pathway under IH conditions. *Berberine* improves liver lipid deposition by modulating the intestinal microbiota to enhance the production of short-chain fatty acids (SCFAs) and activating the AMPK/ACC pathway. Clinical trials have demonstrated that administration of BBR (500 mg three times daily) reduces hepatic lipid content by 32% in patients with non-alcoholic fatty liver disease (). It has been reported that *baicalein* enhances the assembly, stability, and activity of the AMPK holoenzyme while inhibiting ferroptosis in chondrocytes through the induction of AMPKα phosphorylation. Furthermore, AMPKα maintains the expression levels of nuclear factor erythroid 2-related factor 2 (Nrf2) in chondrocytes and facilitates its translocation into the nucleus by promoting Keap1 degradation. Concurrently, Nrf2 upregulates the expression of heme oxygenase-1 (HO-1), thereby suppressing lipid reactive oxygen species (ROS) accumulation in chondrocytes. These findings indicate that *baicalein* mitigates chondrocyte ferroptosis by augmenting the activity of the AMPK/Nrf2/HO-1 signaling pathway, thus alleviating the progression of osteoarthritis (OA) (Wan et al., 2023). Studies demonstrate that curcumin decreases OARSI and Mankin scores in OA rats, suppresses MMP13 expression, promotes Collagen II synthesis, and upregulates mitochondrial autophagy-related proteins (PINK1, Parkin, LC3B). These effects are abrogated by treatment with the AMPK inhibitor (Compound C) or the autophagy inhibitor (CsA). Curcumin restores IL-1β-induced mitochondrial dysfunction, as evidenced by enhanced mitochondrial membrane potential and ATP levels, reduced ROS production, and decreased Ca^2+^ concentration. It activates AMPK phosphorylation and enhances autophagy-related protein expression, confirmed by Western blot and qPCR analyses. The mechanism involves curcumin eliminating abnormal mitochondria, alleviating oxidative stress, and suppressing inflammation to mitigate cartilage degradation (Jin et al., 2022).

#### 3.3.2 TGF-β/Smads pathway

TGF-β1 induces fibrosis through the activation of both classical (Smad-dependent) and non-classical (non-Smad-dependent) signaling pathways, leading to myofibroblast activation, excessive extracellular matrix (ECM) production, and inhibition of ECM degradation. The roles of Smad proteins in regulating fibrosis are multifaceted, encompassing both profibrotic and anti-fibrotic effects, including their involvement in the regulation of mesenchymal transition. Furthermore, there is a complex interplay between TGF-β/Smads and other signaling pathways that modulates fibrotic processes (Meng et al., 2016).

*Resveratrol* suppresses lung metastasis of LoVo cells *in vivo*. Furthermore, *resveratrol* decreases the incidence of lung and liver metastases in orthotopic transplantation models in mice. *In vitro* studies demonstrate that TGF-β1-induced epithelial-mesenchymal transition (EMT) facilitates colorectal cancer (CRC) invasion and metastasis by reducing E-cadherin expression, enhancing Vimentin expression, and activating the TGF-β1/Smads signaling pathway. Conversely, *resveratrol* exhibits a concentration-dependent inhibitory effect on the invasion and migration of LoVo cells, upregulates E-cadherin expression, downregulates Vimentin expression, and suppresses the TGF-β1/Smads signaling pathway (Ji et al., 2015). Previous studies (Ji et al., 2016) have demonstrated that in mice with pulmonary fibrosis, administration of *paeoniflorin* (50, 100 mg kg^-1^·d^-1^) or prednisone (6 mg kg^-1^·d^-1^) significantly decreased the expression of FSP-1 and α-SMA while enhancing the expression of E-cadherin in lung tissue. In A549 cells, stimulation with TGF-β1 induced epithelial-mesenchymal transition (EMT), as evidenced by alterations in cell morphology, increased cell migration capacity, elevated expression of vimentin and α-SMA, increased levels of collagen type I and III, and reduced expression of E-cadherin. Total flavonoids from *Astragalus membranaceus* significantly alleviate alveolar inflammation and collagen deposition in the lung tissue of bleomycin (BLM)-induced pulmonary fibrosis (PF) mouse models, while inhibiting the expression of tumor necrosis factor-α (TNF-α) and transforming growth factor-β1 (TGF-β1) proteins. Notably, the therapeutic efficacy of the total flavonoids group was markedly superior to that of the astragalus polysaccharide group and the astragaloside control group (Xu et al., 2016). Pre-treatment of MRC-5 cells with *matrine* derivatives in a TGF-β1-induced pulmonary fibrosis (PF) model demonstrated that *matrine* derivatives significantly decreased the viability of MRC-5 cells. *Matrine* was found to inhibit the binding of TGF-β1 to its receptor, downregulate the expression levels of Smad2/3, reduce collagen deposition, and block the translocation of cytoplasmic proteins to the nucleus. These findings suggest that *matrine* derivatives may suppress the proliferation and activation of MRC-5 cells via the TGF-β1/Smad signaling pathway, thereby altering the biological morphology of the cells and providing a potential therapeutic approach for pulmonary fibrosis (Li L. et al., 2019).

#### 3.3.3 JAK/STAT pathway

Janus kinases (JAKs) are non-transmembrane tyrosine kinases that are ubiquitously expressed across a wide range of cell types. The cytoplasmic signal transducers and activators of transcription (STATs), as downstream effectors of JAK, play a critical role in cellular signal transduction and transcriptional regulation. The JAK/STAT signaling pathway is implicated in numerous biological processes associated with fibrosis, including inflammatory responses, oxidative stress, and apoptosis. This pathway can be modulated by various cytokines and interferons, such as the pro-inflammatory cytokines IL-4, IL-6, and IL-13, as well as the profibrotic factor TGF-β1. Upon stimulation by these cytokines, the STAT transcription factors are phosphorylated and activated, subsequently transmitting signals to downstream targets. Specifically, members of the JAK family mediate the phosphorylation of STAT1 and STAT3. This activates the JAK/STAT signaling pathway and facilitates the transmission of signals into the nucleus for transcriptional regulation. This process ultimately induces the activation of fibroblasts (Xin et al., 2020; Erdogan et al., 2022; Deimel et al., 2021).

Several studies have confirmed that *Jolkinolide B* (JB), a natural extract derived from a Chinese herb, may exert anti-rheumatoid arthritis (RA) effects via the regulation of Th17 cell differentiation, prolactin signaling pathways, and JAK/STAT signaling pathways. Molecular docking and SPR analyses demonstrated that JB binds efficiently to JAK2. In a study on collagen-induced arthritis (CIA) rats, JB significantly attenuated body weight loss, reduced arthritis indices, paw thickness, and synovial thickness. Therefore, it can be concluded that JB exhibits a novel inhibitory effect on inflammation and bone destruction in CIA rats, potentially through the modulation of the JAK2/STAT3 signaling pathway (Yan et al., 2024). The study demonstrated that *salidroside* significantly ameliorated cecal ligation and puncture-induced sepsis-associated acute lung injury in pulmonary fibrosis (PF) mice. It effectively reduced the levels of inflammatory cytokines, including IL-6, IL-4, and IL-10, which are released during acute lung injury. Furthermore, *salidroside* downregulated the expression of JAK2 and STAT3 proteins. Additionally, *salidroside* inhibited the expression of fibrosis-related proteins, such as vimentin, α-SMA, FN, and Col I, in lung tissue. These findings suggest that *salidroside* may exert an anti-pulmonary fibrosis (PF) effect by suppressing the JAK2/STAT3 signaling pathway, thereby attenuating the inflammatory response and promoting collagen fiber degradation (Guo et al., 2021).

### 3.4 Keap1-Nrf2/ARE pathway

One of the most critical defense mechanisms against oxidative and/or electrophilic stress is the Keap1-Nrf2-ARE (Kelch-like ECH-associated protein 1-nuclear factor erythroid 2-related factor 2-antioxidant response element) pathway. This pathway plays a pivotal role in regulating cellular responses to stress and is intricately linked to various inflammatory diseases, such as cancer, neurodegenerative disorders, cardiovascular conditions, and aging processes (Lu et al., 2016). The Keap1/Nrf2/ARE signaling system, which is sensitive to redox changes, plays a crucial role in maintaining cellular homeostasis under conditions of stress, inflammation, carcinogenesis, and pro-apoptotic signaling, thereby positioning it as a promising pharmacological target (Tu et al., 2019).

*Ginkgolide B* activates the Nrf2 pathway, leading to the upregulation of HO-1 and SOD2, thereby alleviating oxidative stress induced by Aβ. In the Alzheimer’s disease model, hippocampal neuronal apoptosis was significantly reduced by 64% (). The bupleurum extract, *Saikosaponin*, has been demonstrated *in vivo* to prevent experimental sepsis through the inhibition of NOD2-mediated NF-κB activation. Additionally, it exerts a critical regulatory effect on LPS-induced acute lung injury by blocking the NF-κB and NLRP3 inflammasome signaling pathways (Zhao et al., 2015) (Du et al., 2018). It has been confirmed that PF significantly suppresses high glucose-induced oxidative stress in Schwann cells by reducing ROS and MDA levels while enhancing GST and GPX activities. Additionally, PF facilitates the dissociation of Nrf2 from Keap1 and upregulates the Nrf2/antioxidant response element (ARE) pathway (Yang et al., 2016).

### 3.5 BDNF/TrkB pathway

Brain-derived neurotrophic factor (BDNF) plays a critical role in neuronal plasticity, learning, and memory through the activation of the full-length TrkB receptor (TrkB-FL). Abnormal truncation of TrkB mediated by calpain has been reported to result in dysregulation of BDNF/TrkB signaling pathways, which is closely associated with cognitive impairment in various neurodegenerative diseases. Calpain, a Ca2+-dependent protease, exhibits excessive activation under pathological conditions, contributing to neuronal death (Qiu et al., 2020). BDNF, along with its receptors TrkB and p75 neurotrophic factor receptor (p75), plays a critical role in regulating dendritic growth in the central nervous system (CNS). Following BDNF binding, TrkB and p75 are internalized into endosomes and continue to transmit signals within the cell body, dendrites, and axons (Moya-Alvarado et al., 2023).

A post-stroke depression (PSD) rat model was successfully established through middle cerebral artery occlusion (MCAO) combined with chronic unpredictable mild stress (CUMS). Subsequently, the rats were treated with echinoside (ECH). ECH was found to enhance the transcriptional activity of brain-derived neurotrophic factor (BDNF), activate the BDNF/TrkB signaling pathway, and mediate a coordinated response against oxidative stress and apoptosis. Consequently, the PSD symptoms in the rats were significantly alleviated (Yang Z. et al., 2024). In addition, several animal studies (Liu et al., 2023) have demonstrated that TAN IIA treatment alleviates depression-like behavior and cognitive deficits induced by chronic unpredictable mild stress (CUMS) in rats. Furthermore, TAN IIA treatment has been shown to reverse the impact of CUMS on dendritic complexity and γ-aminobutyric acid (GABA) levels within the hippocampus and prefrontal cortex. Rats exposed to CUMS exhibited decreased BDNF and phosphorylated TrkB levels, upregulated GABA transporter 1 (GAT1) expression, and reduced synapsin expression in the hippocampus. Notably, TAN IIA treatment significantly mitigated these effects associated with CUMS exposure. The aqueous extract of Polygala tenuifolia (YT) constitutes the primary component of frequently utilized Traditional Chinese Medicine formulations (TCMFGs), exhibiting significant neuroprotective effects (Cao et al., 2012). YT exhibits antidepressant effects in rats displaying CUMS-induced depression-like behavior, promoting recovery through enhanced hippocampal neurogenesis and activation of the BDNF signaling pathway (Jiang et al., 2021).

## 4 Limitations and challenges of the current

### 4.1 Insufficient research depth

1) The imbalance between monomer and single flavor: Previous studies have primarily focused on individual metabolites, such as berberine and tetramethylpyrazine; however, the mechanisms of single-flavor and compound concentrated granules remain less explored. Although individual metabolites can clearly identify specific targets (such as berberine inhibiting the TLR4/NF-κB pathway), the synergistic nature of traditional Chinese medicine’s “multi-metabolites-multi-targets-multi-pathways” paradigm is often overlooked (Lai et al., 2024). Studies (Qiu et al., 2019) have demonstrated that, in addition to berberine, alkaloids such as coptisine and jatrorrhizine in Coptis concentrated granules may enhance anti-inflammatory effects via epigenetic regulation, such as DNA methylation modification. However, the precise mechanisms remain to be elucidated. This serves as an illustrative example of how the underlying mechanisms of many traditional Chinese medicine metabolites are still poorly understood, thus highlighting the need for more in-depth investigations. In addition, gaps in compound studies, the synergistic effects of compound concentrated granules, such as Siwu decoction and Buyang Huanwu decoction, have yet to be subjected to systematic analysis. For instance, co-decocting *Astragalus membranaceus* and Angelica sinensis may lead to the formation of supramolecular polymers, including astragalus polysaccharide-Angelica lactone complexes. However, the underlying mechanisms of synergistic regulation of the PI3K/Akt and HIF-1α pathways remain poorly understood (Wu et al., 2021).
2) Pathway interaction complexity: The PI3K/Akt and mTOR pathways are highly interactive in the regulation of tumor metabolism; however, the majority of existing studies have focused on isolating and analyzing individual pathways. For instance, while *salidroside* has been shown to inhibit Akt/mTOR signaling, whether it indirectly regulates mTOR via the AMPK-SIRT1 axis remains a subject of debate. The integrated regulation of the immune-metabolism-fibrosis network by concentrated particles has not been extensively investigated. For instance, Panax notoginseng saponins (PNS) suppress both the TGF-β/Smads and PDGF/ERK pathways; however, the mechanisms by which they exert synergistic effects via the YAP/TAZ mechanosignaling pathway warrant further exploration (Wu et al., 2021).

### 4.2 Unclear component interactions

1) The equivalence dispute between separate decoction and combined decoction: Differences in chemical reactions: Acid-base neutralization, complex precipitation, oxidation polymerization, and other reactions may occur during the co-decoction of compound concentrated granules, leading to the formation of new substances. For instance, the Sini decoction, which consists of aconitine, dried ginger, and licorice, demonstrates a synergistic effect when its metabolites are combined. Specifically, aconitine forms a complex with glycyrrhizic acid, thereby reducing toxicity while enhancing the cardiotonic effect. However, this effect could not be reproduced following the frying and mixing process (Ding et al., 2023). The combined decoction of *Coptidis rhizoma* and *Tetradium ruticarpum* generated a *Coptidine*- *Tetradium* lactone complex, which exhibited an inhibitory effect on NF-κB activity that was 2.3 times greater than that of the simple decoction mixture (Nan et al., 2022).
2) insufficient quantitative assessment of synergistic and antagonistic effects: After co-decoction of *Astragalus* and *Zedoary* turmeric concentrated granules, astragaloside IV and β-elemene synergistically inhibited the activity of liver cancer stem cells via the PI3K/Akt-Hippo/YAP signaling pathway (synergy index CI = 0.62). On the contrary, ginsenoside Rg1 demonstrated oxidative antagonistic effects when co-decocted with pentolinic acid, leading to a 52% reduction in antiplatelet activity (Qin, 2006).

### 4.3 Standardization and the bottleneck in clinical translation

1) According to the heterogeneity of medicinal materials, the effective metabolites of the same medicinal material in different producing areas and batches are significantly different. In addition, low-temperature dynamic countercurrent extraction (<60°C) can retain thermosensitive metabolites such as *tetramethylpyrazine*, but this process requires high equipment, so the extraction process needs to be optimized (Zhang et al., 2004).
2) The evidence of clinical translation is weak: There is a notable scarcity of cross-disease data in current research. Existing clinical studies predominantly concentrate on a limited number of areas, such as liver disease and cancer, with insufficient large-sample evidence for cardiovascular and neurodegenerative diseases. For instance, in the cardiovascular domain, the mechanism by which *Salvia miltiorrhiza* granules improve myocardial ischemia has been investigated primarily through animal models (n = 50), lacking robust multicenter randomized controlled trial (RCT) data to support these findings (Cheng and Jiang, 2005; Zhu, 2006). It is important to note that pediatric applications also present certain limitations. The absence of standardized dosing guidelines necessitates reliance on experiential adjustments for children’s medication, and 90% of concentrated particles lack labeling for pediatric dosing regimens (Zhuang et al., 2014).

### 4.4 Methodological limitations in experimental design

1) Overreliance on Single-Component Analysis: Most studies focus on isolated metabolites (e.g., berberine, tetramethylpyrazine) rather than the holistic “multi-component-multi-target” nature of TCMFG. While this clarifies specific mechanisms (e.g., berberine inhibits TLR4/NF-κB), it neglects synergistic interactions. For example, Coptis Rhizoma granules combined with Scutellaria baicalensis polysaccharides enhance berberine bioavailability by 1.8-fold and reduce hepatotoxicity by 35% compared to berberine alone (Wang K. et al., 2024). Such synergy, central to TCM theory, requires systematic exploration via metabolomics and network pharmacology (Qiu et al., 2019).
2) Low Clinical Relevance of Preclinical Models: Widely used animal models (e.g., MIA-induced osteoarthritis in rats) inadequately mimic human disease complexity. These models primarily reflect structural cartilage damage but fail to replicate chronic inflammation or immune dysregulation seen in patients (Li et al., 2023). Emerging tools like patient-derived organoids or humanized mouse models could bridge this gap (Zhang and Zhan, 2025).
3) Unoptimized and Arbitrary Dosing: Many studies lack pharmacokinetic (PK) rationale for dose selection. For instance, *salidroside* anti-tumor dose (20 mg/kg in mice) lacks human-equivalent scaling, complicating clinical extrapolation (Wu et al., 2021). Future work should integrate physiologically based pharmacokinetic (PBPK) modeling and dose-response studies to establish therapeutic windows.
4) Inadequate Experimental Controls: A significant proportion of studies omit essential controls. For example, anti-inflammatory evaluations of TCMFG rarely include positive controls (e.g., dexamethasone for NF-κB inhibition), making it impossible to assess comparative efficacy (Zhang and Zhan, 2025). Negative controls (e.g., vehicle-treated groups) are also frequently underspecified, raising concerns about result specificity.

## 5 Future research directions

### 5.1 Multi-omics integrated analysis

Current studies predominantly focus on single-omics approaches (e.g., transcriptomics or metabolomics). However, to fully characterize the multi-target nature of traditional Chinese medicine metabolites, it is necessary to integrate metabolomics, proteomics, spatial transcriptomics, and single-cell sequencing technologies to construct a multi-dimensional regulatory network.

1) Combined metabonomics and proteomics analysis enables the systematic investigation of spatiotemporal and dynamic interactions between host metabolites (e.g., short-chain fatty acids, bile acids) and key proteins (e.g., PI3K/Akt, mTOR) following the intervention of concentrated particles. For instance, the synergistic effect of Astragalus and shikonin has been demonstrated to improve diabetic ulcers by modulating tryptophan metabolism (via the IDO1 pathway) and activating the PI3K-Akt signaling pathway (Li et al., 2017; Luo et al., 2020).
2) Spatial transcriptomic localization: When combined with the organoid model, spatial transcriptomics analyses have revealed the differential regulation of the NF-κB and Hippo/YAP signaling pathways by concentrated particles originating from distinct regions of the hepatic lobules, such as the central venous area and the portal venous area. (An, 2019). In addition, this study elucidated the protective effects of *Shexiang Baoxin Pill* (SBP) against myocardial ischemia-reperfusion (I/R) injury. SBP markedly upregulated genes associated with myocardial contractility (Tnnt2 and Myh6), enhanced left ventricular ejection fraction (LVEF increased from 51.74% to 58.02% in the I/R group), and promoted angiogenesis via the elevated expression of Npr3 and Nppb in endothelial cells. Furthermore, SBP suppressed fibroblast activation (collagen deposition reduced by 68%), downregulated TGF-β/Smad pathway-mediated fibrosis markers (Col1a1, Acta2), and induced macrophage polarization toward an anti-inflammatory phenotype (Arg1↑, NOS2↓), thereby optimizing the immune microenvironment. Additionally, multi-omics data revealed that SBP improved energy metabolism and alleviated oxidative stress damage through the AMPK/mTOR pathway, which is consistent with clinically observed reductions in serum LDH and cTnI levels (by 23.5% and 61.2%, respectively) (Lin et al., 2023).
3) Single-cell immune microenvironment analysis: Single-cell RNA sequencing (scRNA-seq) was employed to investigate the phenotypic switching of immune cells, including Treg and M2 macrophages, under the influence of compound intervention. Additionally, this study aims to elucidate the remodeling of the tumor microenvironment mediated by the Bupleural-Baicalicaria pair via the IL-6/STAT3-TGF-β axis (Wang et al., 2020).

### 5.2 Mechanism exploration of compound synergy: transitioning from “composition superposition” to “supramolecular synergy”

Most of the existing studies focus on single drug or single component, while the “co-decoction synergistic effect” of compound concentrated granules requires three aspects: molecular interaction, dynamic metabolism and physicochemical properties.

1) The molecular interaction mechanism can be elucidated using cryo-electron microscopy (Cryo-EM) and nuclear magnetic resonance (NMR), thereby confirming that the astragalus polysaccharide-angelicolactone supramolecular polymer generated by Siwu decoction enhances anti-inflammatory activity via the TLR4/MyD88-NF-κB signaling pathway. Furthermore, in the context of dynamic metabolic interactions, real-time metabolic flux analysis (e.g., employing 13C labeling technology) can be utilized to monitor the inhibitory effect of *baicalin* on the metabolic transformation of *saikosaponin* within the gut microbiota, thereby improving its bioavailability (Mao et al., 2024; Chang et al., 2016). In addition, Yang et al. (Yang L. et al., 2024) employed cryo-electron microscopy (Cryo-EM) to verify the formation of Mahuang-Fuzi (MGF-SA) supramolecular polymers, which demonstrate enhanced anti-inflammatory activity in comparison to physical mixtures (MIX). These supramolecular assemblies originate from dynamic non-covalent interactions between alkaloids (e.g., ephedrine derived from Ephedra sinica) and polysaccharides (e.g., astragalus polysaccharides extracted from *Astragalus membranaceus*) during low-temperature concentration processes. Structural analysis indicated that MGF-SA forms stable helical polymers via hydrogen bonding and π-π stacking interactions, facilitating prolonged retention within inflammatory tissues and sustained release of bioactive metabolites.

Mechanistically, MGF-SA suppresses the nuclear translocation of NF-κB p65 and downregulates pro-inflammatory cytokines (e.g., TNF-α, IL-6) by modulating the TLR4/MyD88-NF-κB pathway. This effect is 2.3-fold stronger than that of individual metabolites or physical mixtures, as demonstrated by reduced NO production (IC_50_ = 12.5 μM vs 28.7 μM for MIX) in RAW264.7 cells (Yang L. et al., 2024). The enhanced activity is attributed to the supramolecular structure’s ability to simultaneously block multiple inflammatory checkpoints, including MD2-TLR4 binding and IκBα phosphorylation, thereby amplifying pathway inhibition.

2) In the context of quantifying synergy/antagonism effects, an Herbal Synergy Index (HSI) can be established to measure interactions between metabolites using a systems pharmacology model. To systematically evaluate supramolecular synergy, the Herbal Synergy Index (HSI) has been introduced. This index integrates key parameters such as structural stability, bioavailability, and multi-target engagement to provide a comprehensive assessment. For instance, the HSI for MGF-SA was determined to be 2.1, which signifies robust synergistic effects. In contrast, physical mixtures yielded an HSI of 0.9, indicating antagonistic interactions likely caused by competitive binding among metabolites (Yang L. et al., 2024). In addition, when Rhizoma coptidis and evotaecarpine are combined in a decoction, the HSI for berberine and evotaecarpine is 1.8 (indicating synergy), whereas the HSI for separate decoctions is only 0.7 (indicating antagonism) (Guo et al., 2023). Furthermore, the development of organoid chips to simulate dynamic metabolism of complex metabolites during intestinal and hepatic circulation represents a critical direction. This includes mechanisms such as Shenqi Jiangtang granules acting via the intestinal epithelial L-cell GLP-1R-PKA pathway and the hepatic AMPK pathway to regulate glucose levels (Wang et al., 2020).

### 5.3 The connection between clinical and basic research: from “evidence fragments” to “precision medicine”

Current clinical studies are predominantly restricted to small-sample, single-disease observations. To validate the multi-pathway regulatory effects of concentrated particles, large-scale randomized controlled trials (RCTs) integrated with real-world data (RWD) are essential. To develop an Herbal Pathway Activity Score (HPAS) and quantify the individual pathway status of patients using multi-omics data for guiding treatment decisions. If the HPAS of diabetic microcirculation disorder patients indicates low PI3K-Akt signaling activity, the use of Shenqi Jiangtang granule is preferred over its monomer metabolites. In addition, real world evidence (RWE) database was established to include long-term drug safety data (such as 10-year follow-up) and identify rare adverse reactions (such as drug resistance gene transfer in intestinal flora induced by Coptis chintis concentrate particles) (Xu et al., 2024).

## 6 Conclusion

TCMFGs exemplify the convergence of traditional medicine and modern pharmacology. Through standardized extraction (e.g., dynamic countercurrent) and multi-target synergy (e.g., berberine-TLR4, tetramethylpyrazine-MAPK), they offer novel therapeutic strategies for complex diseases. However, translational success demands rigorous clinical validation, advanced models, and quantifiable synergy metrics like the Herbal Synergy Index (HSI). Future research must bridge reductionist single-metabolite approaches with TCM’s holistic paradigm to realize precision medicine potential.
